# Evidence for Epithelial-Mesenchymal Transition in Cancer Stem Cells of Head and Neck Squamous Cell Carcinoma

**DOI:** 10.1371/journal.pone.0016466

**Published:** 2011-01-27

**Authors:** Chao Chen, Yan Wei, Michael Hummel, Thomas K. Hoffmann, Manfred Gross, Andreas M. Kaufmann, Andreas E. Albers

**Affiliations:** 1 Department of Otolaryngology, Head and Neck Surgery, Charité-Universitätsmedizin Berlin, Berlin, Germany; 2 Department of Pathology, Charité-Universitätsmedizin Berlin, Berlin, Germany; 3 Department of Otolaryngology, Head and Neck Surgery, University of Essen, Essen, Germany; 4 Department of Audiology and Phoniatrics, Charité-Universitätsmedizin Berlin, Berlin, Germany; 5 Clinic for Gynecology, Charité-Universitätsmedizin Berlin, Berlin, Germany; University of Pennsylvania, United States of America

## Abstract

Initiation, growth, recurrence, and metastasis of head and neck squamous cell carcinomas (HNSCC) have been related to the behavior of cancer stem cells (CSC) that can be identified by their aldehyde-dehydrogenase-isoform-1 (ALDH1) activity. We quantified and enriched ALDH1^+^ cells within HNSCC cell lines and subsequently characterized their phenotypical and functional properties like invasion capacity and epithelial-mesenchymal transition (EMT). Spheroid culture enriched CSC from five HNSCC cell lines by up to 5-fold. In spheroid-derived cells (SDC) and the parental monolayer-derived cell line ALDH1, CD44, CD24, E-Cadherin, α-SMA, and Vimentin expression was compared by flow-cytometry and immunofluorescence together with proliferation and cell cycle analysis. Invasion activity was evaluated by Matrigel assay and expression of stemness-related transcription factors (TF) Nanog, Oct3/4, Sox2 and EMT-related genes Snail1 and 2, and Twist by real-time PCR. All cell lines formed spheroids that could self-renew and be serially re-passaged. ALDH1 expression was significantly higher in SDC. ALDH1^+^ cells showed increased colony-formation. The proportion of cells with a putative CSC marker constellation of CD44^+^/CD24^−^ was highly variable (0.5% to 96%) in monolayer and spheroid cultures and overlapped in 0%–33% with the CD44^+^/CD24^−^/ALDH1^+^ cell subset. SDC had significantly higher invading activity. mRNA of the stemness-related genes Sox2, Nanog, and Oct3/4 was significantly increased in SDC of all cell lines. Twist was significantly increased in two while Snail2 showed a significant increase in one and a significant decrease in SDC of two cell lines. SDC had a higher G0 phase proportion, showed high-level expression of α-SMA and Vimentin, but significantly decreased E-Cadherin expression. HNSCC-lines harbor potential CSC, characterized by ALDH1 and stemness marker TF expression as well as properties like invasiveness, quiescence, and EMT. CSC can be enriched by anchorage-independent culture techniques, which may be important for the investigation of their contribution to therapy resistance, tumor recurrence and metastasis.

## Introduction

HNSCC accounts for approximately 6% of all cancer cases and for about 650,000 new cases and 350,000 deaths worldwide each year [1], [2], [3]. Advances in therapy have improved quality of life, but survival rates have remained unchanged over the past decades. Mortality from this disease remains high because of the development of distant metastases and the emergence of local and systemic recurrences resistant to chemo- and radiotherapy. It is therefore essential to develop a deeper understanding of the biology of this disease in order to develop more effective therapeutic approaches.

Evidence has recently been accumulating to support the hypothesis that tumors contain a small subpopulation of cells called cancer stem cells (CSC), which exhibit self-renewing capacities and are responsible for tumor maintenance and metastasis [4]. CD44^+^/CD24^−^cells have been firstly proposed to exhibit CSC properties in breast cancer [5].

Subsequently, CD133 was found to identify CSC in brain tumors [6], colorectal carcinoma [7], and pancreatic carcinoma [8]. In HNSCC, Prince et al. first demonstrated that a CD44^+^ population of cells possesses the properties of CSC [9], but relatively high numbers of these cells (>5,000 cells) were needed to generate new tumors in immunodeficient mice indicating either a low frequency of CSC or a low specificity of CD44 as CSC-marker in HNSCC. The latter hypothesis is supported by the observation that CD44s and CD44v6 expression does not distinguish normal from benign or malignant epithelia of the head and neck. CD44s and CD44v6 were abundantly present in the great majority of cells in head and neck tissues, including carcinomas [10]. Thus, the identification of more specific CSC markers for HNSCC is desirable. Recently, high aldehyde dehydrogenase 1 (ALDH1, also known as ALDH1A1) activity was shown to identify the CSC in HNSCC and other epithelial cancers [11], [12], [13], [14], [15]. However, in breast cancer the ALDH1^+^ population shows a surprisingly small overlap with the previously described CD44^+^/CD24^−^ phenotype of only 0.1–1.2%. Interestingly, in breast cancer the cells bearing both phenotypes appeared to be highly tumorigenic, being able to generate tumors from as few as 20 cells [15]. It remains to be determined if the same phenotypic pattern of stem cells in HNSCC is associated with a similar tumorigenic potential.

Non-adherent sphere assays are increasingly being used to evaluate stem cell activity in normal tissue and putative CSC. The neurosphere is the best-studied sphere assay. Central nervous system cells grown on nonadherent surfaces give rise to neurospheres that have the capacity for self-renewal and can in principal generate all the cell types of the brain [16], [17]. The capacity for repeated generation of neurospheres from single cells is generally viewed as evidence of self-renewal [18]. Recently, it was described that spheroid-derived cells (SDC) from gliosarcoma rat cell lines possess CSC capacity [19]. So far, it is unknown whether spheroid cultures of HNSCC can enrich for CSC.

Normal hematopoietic stem cells are able to self-renew and give rise to all blood cell types. They are non- or very slow-dividing cells and reside within bone marrow niches in a dormant condition. The ability to maintain this non-dividing condition, or in other terms, stay in the G0 phase of cell cycle, is called quiescence [20]. It has been proposed that CSC share many qualities with normal stem cells since the vast majority of them also should stay quiescent and be able to self-renew. Because a majority of anti-cancer drugs target actively dividing (cycling) cells, quiescent CSC remain alive and may be the cause for relapses and progression of cancer. Ki-67 antigen is the prototypic cell cycle related nuclear protein, expressed by proliferating cells in all phases of the active cell cycle (G1, S, G2 and M phase), but it is absent in resting (G0) cells.

Another capability of CSC is to perform epithelial–mesenchymal transition (EMT), a key step during embryogenesis [21], [22], [23] and wound healing [24]. Recent evidence suggests that genetic programs relevant for EMT are also transiently activated in epithelial cancers playing a role in cancer progression, through which epithelial cancers invade tissues and metastasize. Although the EMT program is necessary for normal development, the aberrant activation of EMT contributes to various pathologic conditions, including fibrosis and carcinoma progression [25], [26]. During EMT epithelial cells break down cell-cell and cell-extracellular matrix contacts and migrate to other locations in the body [27]. During cancer progression, EMT seems to provide cancer cells with the capacity to infiltrate the surrounding tissue and ultimately metastasize to distant sites [28]. Recently, it has been reported that the induction of EMT in differentiated immortalized human mammary epithelial cells led to the acquisition of the CD44^+^/CD24^−^ stem cell phenotype [29]. Moreover, it was shown that these putative CD44^+^/CD24^−^ CSC isolated from neoplastic human breast tissues expressed high mRNA levels encoding the EMT-associated markers Snail1 (SNA), Snail2, and Twist. Malignant tumors consist of cancer cells and tumor-associated host cells, with the latter attracting more interest recently because of their participation in tumor invasion and metastasis, and therapeutic response [30], [31]. Myofibroblasts are the most important components of the tumor stroma. Nevertheless, the origin of these cells remains controversial so far. The expression of alpha-smooth muscle actin (α-SMA) is considered the marker of the fully differentiated myofibroblasts. Emerging evidence shows that myofibroblasts can be derived from the epithelial (tumor) cells via EMT [32], [33]. This notion is supported by the observation that compact spheroids formed by ovarian cancer cells in ascites display contractile behavior, possess high invading capacity in vitro and the expression of α-SMA, which is also associated with high contractile capacity [34].

In the present work, we tested if anchorage-independent cell culture techniques allow the generation of spheroid-cultures and if these cultures were enriched for cells with functional and phenotypic properties characterizing CSC of HNSCC. Here, we provide evidence that myofibroblasts can be derived from HNSCC using a spheroid cell culture model that enriches for CSC-like cells as characterized by a high proportion of ALDH1 positivity, proliferative quiescence, and invasive capacity.

## Results

### HNSCC cell lines contain cells with self-renewing capacities that form tumor spheroids

**Figure 1:**
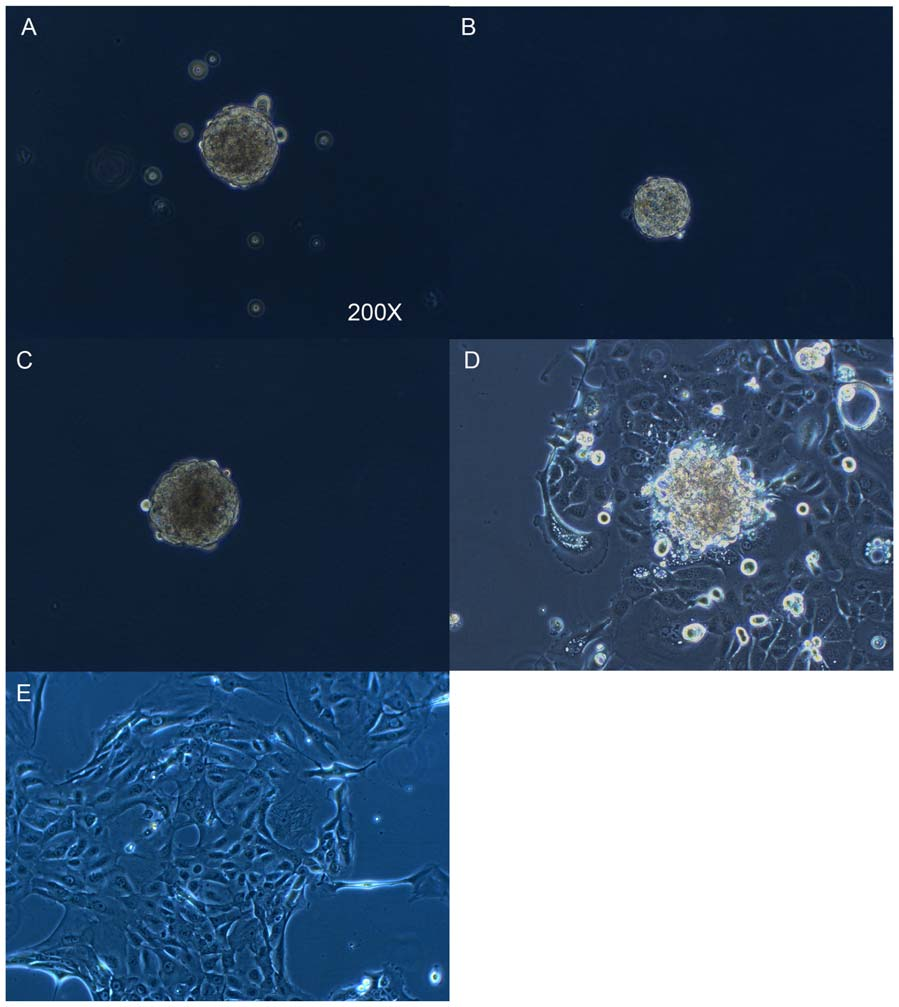
Spheroids generated from HNSCC lines in serum-free medium have self-renewing capacity. Representative pictures of the cell line UD-SCC1 are shown. (**A**) The first generation of spheroids. (**B**) A subspheroid formed after seeding at a concentration of 1,000 cells/ml. (**C**) A spheroid formed in the 21st generation. (**D**) Spheroid adhering and growing to confluence after replating into flasks coated for tissue culture. (**E**) Parental cell line grown permanently as a monolayer culture.

Several different approaches have been used to identify CSC. The strategy used for our experiments was the in vitro spheroid colony formation method. We investigated the ability of five HNSCC-derived cell lines (UD-SCC1, UT-SCC22, UM-SCC11B, UT-SCC9, UT-SCC24A) to grow anchorage independently as spheroid-cultures. Cells were plated at a density of 20,000/ml. After in vitro culture for 5–10 days in serum-free medium under nonadherent conditions, all investigated cell lines formed spheroids, ranging from 50 to 500 cells per spheroid (Figure 1A). The self-renewing capacity of these tumor spheroids was assessed using the method published by Ghods et al [19]. Briefly, the spheroids were collected and dissociated into a single cell suspension that was subsequently plated at a clonal density of 1,000 cells per ml. Tumor subspheroids ranging from 20 to 40 cells were evident after 10 to14 days (Figure 1B, C). In contrast, the parental monolayer cell cultures grown under the same conditions did not form spheroids if plated at this low concentration even after 21 days. When the spheroids were transferred back to a regular tissue culture flask coated for monolayer cell culture, the spheroids adhered to the flask and cells grew out from the spheroid and formed a confluent monolayer. The phenotype of these cells was identical to the parental cell lines (Figure 1D).

### Phenotypic characterization of SDC

The expression of the putative stem cell marker ALDH1 in SDC and the respective parental monolayer cell line used as control was compared to the CD44^+^/CD24^−^ expressing population by multicolor FACS-analysis.

**Figure 2:**
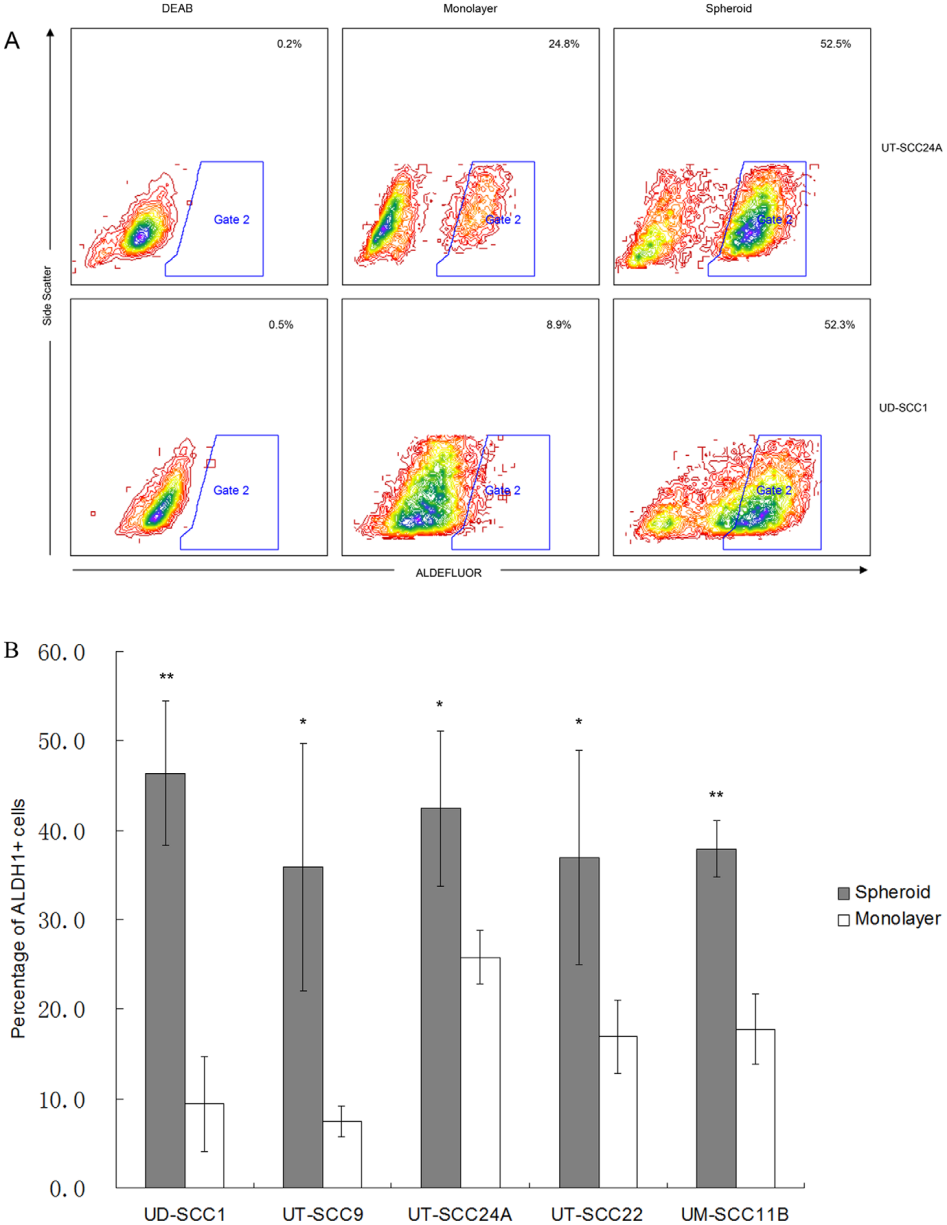
ALDH1 expression in parental cells and spheroids. (**A**) Representative FACS-analysis of two cell lines after Aldefluor staining. Comparison of frequencies of ALDH1 positive cells in monolayer to SDC and DEAB control. DEAB is a specific inhibitor of ALDH1. (**B**) The results represent the expression of ALDH1 in cells derived from spheroid cultures (hedged columns) compared to monolayer cells (open columns). ALDH1 expression of SDC is generally significantly enhanced (A, B). UD-SCC22, UT-SCC24A, and UM-SCC11B displaying a partial mesenchymal phenotype have already relatively high ALDH1 expression in parental cell lines. (** p<0.01, * p<0.05).

Interestingly, all SDC had an increased number of ALDH1 positive cells as compared to parental cell lines (Figure 2). The highest proportion of ALDH1^+^ cells was found in spheroids generated from the UD-SSC1 cell line (46.4±8.1%) which was 5-fold higher than the corresponding parental cell line (9.4±5.3%).

**Figure 3:**
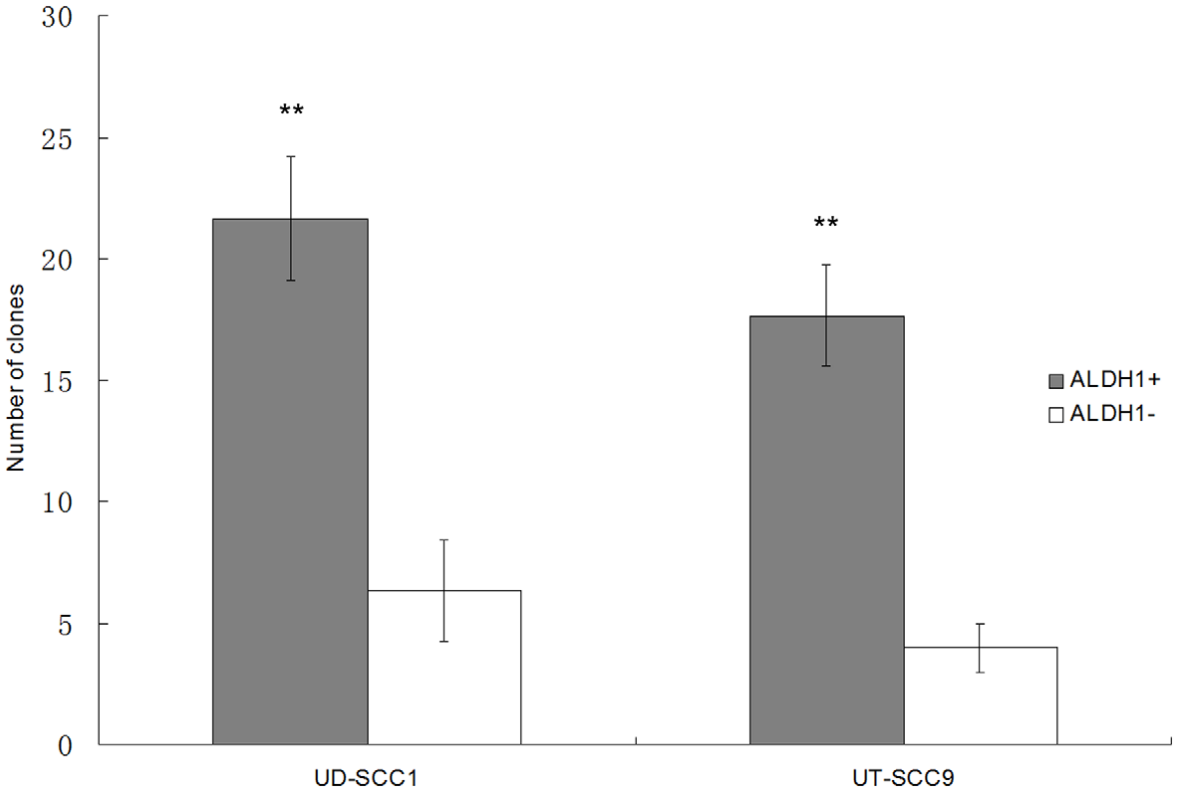
Clone formation assay with FACS-sorted and cloned cells. Two thousand ALDH1-sorted cells were seeded and after 14 days, the colonies that formed were quantified. The ALDH1^+^ subpopulation in UD-SCC1 and UT-SCC9 cell lines have a higher clone formation efficiency as compared to the ALDH1^−^ subpopulation (** p<0.01).

Tumor cells with a CD44^+^/CD24^−^ phenotype were shown to have CSC properties in a variety of solid tumors [4]. We therefore also investigated comparatively the CD44 and CD24 expression in HNSCC cell lines and found that the proportion of CD44^+^/CD24^−^ cells is highly variable (0.5–97%). The overlap with ALDH1^+^ cells was small (0–2.2%) except for the two cell lines UM-SCC11B and UT-SCC22 which had an overlap of about 33% and were composed to over 95% of CD44^+^/CD24^−^ cells. Interestingly, the proportion of CD44^+^/CD24^−^ cells was not consistently enriched by the spheroid culture method (Table 1). This result raised the question how well ALDH1 is suited for the identification of cells with CSC properties in HNSCC. To approach this question, ALDH1 positive and negative cells from the spheroids generated from the cell lines UT-SCC9 and UD-SCC1 were separated by FACS sorting. Subsequently, their capacity for colony formation was assessed. UT-SCC9 and UD-SCC1 were chosen because as compared to the other cell lines they generated more spheroids with higher enrichment of ALDH1^+^ cells. The data show that ALDH1^+^ cells can form 3 to 4 times more clones than ALDH1^−^ cells (Figure 3). Light microscopic observation after 2 weeks showed that the clones formed by ALDH1^+^ cells on average contained 197 (197±47) cells compared with 33 (33±16) cells in clones generated from ALDH1^−^ cells (p<0.01). Our data also show that single ALDH1^+^ cells can significantly better regenerate a spheroid in an anchorage-independent culture with serum-free medium complemented with bFGF and EGF (UT-SCC9: 17.1%, UD-SCC1: 19.3%), whereas under the same conditions single ALDH1^−^ cells regenerated only in one case a spheroid. (Table 2, Figure S1).

**Table 1:** CD44^+^/CD24^−^ phenotype is highly variable in HNSCC and does not consistently overlap with the ALDH1^+^ subpopulation.

Frequency (%)	UD-SCC1	UT-SCC9	UT-SCC24A	UT-SCC22	UM-SCC11B
CD44^+^/CD24^−^ in monolayer cultures	0.44±0.13	0.11±0.07	0.98±0.2	95.27±0.8	95.4±1.25
CD44^+^/CD24^−^ inspheroid cultures	0.96±0.71	3.28±3.54	0.88±0.19	95.17±1.46	96.47±1.62
ALDH1+/CD44^+^/CD24^−^ in monolayer cultures	0.11±0.1	0.08±0.13	0.36±0.33	19.07±1.17	17.37±3.33
ALDH1+/CD44^+^/CD24^−^ in spheroid cultures	0.66±0.52	2.22±2.61	0.53±0.41	33±10.66*	33.4±3.04**

(Data were stated as: mean±standard deviation, % = proportion of positive cells, *p<0.05, **p<0.01).

**Table 2:** Single ALDH1^+^ cells show higher clone formation activity than ALDH1^−^ cells.

Spheroid-formation	Name of cell line			
	UT-SCC9 ALDH1-	UT-SCC9 ALDH1+	UD-SCC1 ALDH1-	UD- SCC1 ALDH+
Yes	1	19	0	23
No	135	92	104	96
Sum	136	111	104	119
Formation Rate	0.7%	17.10%*	0%	19.30%**

Analyzed by chi-square test,

*p<0.05,

**p<0.01.

In summary, SDC generated from HNSCC lines express high levels of the putative CSC marker ALDH1, form significantly more clones, which also significantly regenerate more often into spheroids then ALDH1^−^ cells and have a varying overlap with the CD44^+^/CD24^−^ population.

### SDC show increased invading capacity in vitro

Central to the definition of CSC is the capability to initiate and drive the growth of the primary tumor and of invasion and metastasis. A close relationship between the percentage of cells with CSC phenotype (CD44^+^/CD24^−^) in the primary tumor and the development of metastasis in breast-cancer was recently reported [35]. Also, indication for the metastatic potential of cells with CSC-phenotype in breast cancer comes from the observation that breast cancer cells detected in the bone marrow predominantly showed this phenotype [36].

**Figure 4:**
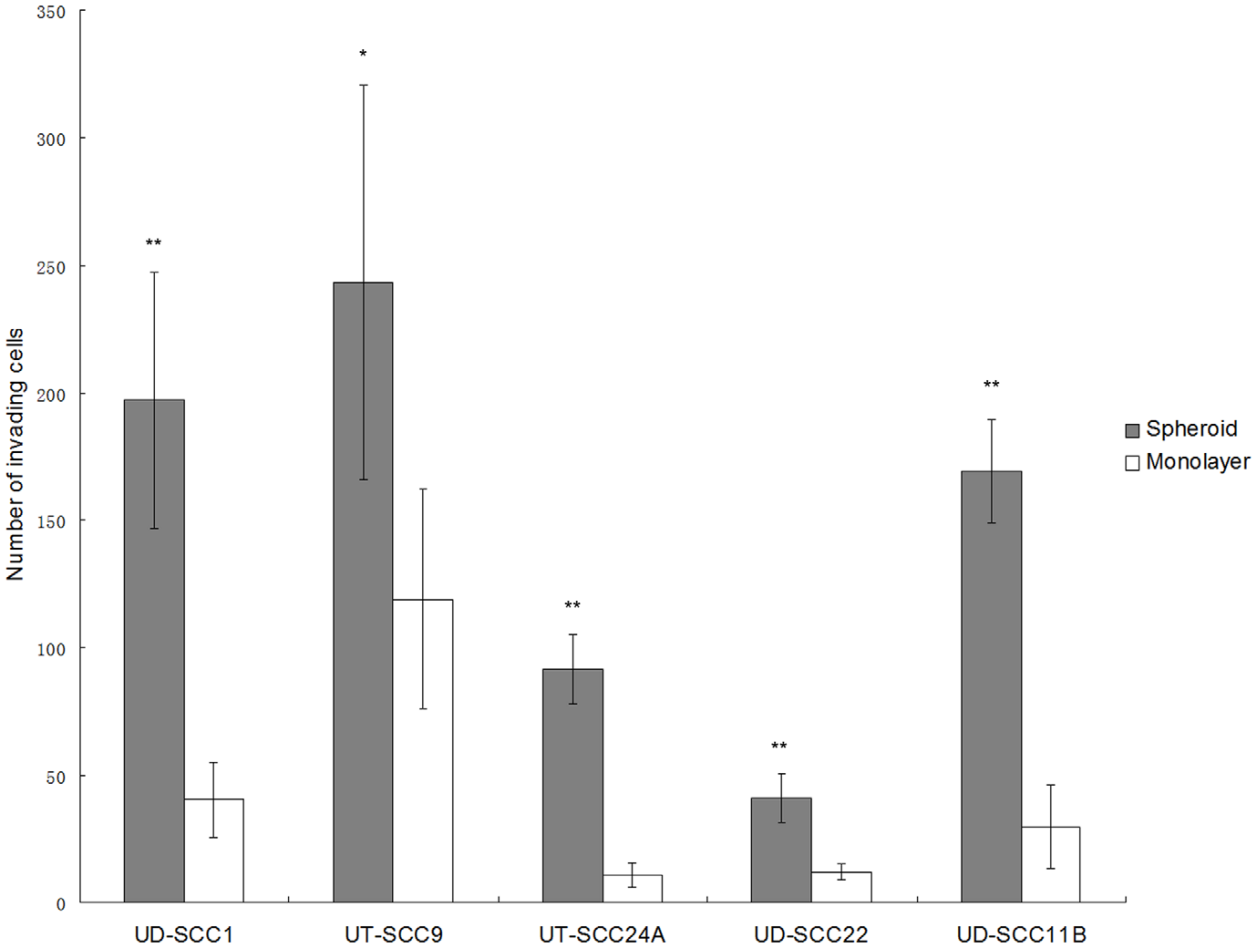
ECM invasion assay. Matrigel invasion chambers were used to compare the invading capacity between cells derived from spheroids (hatched columns) or monolayer (open columns). SDC from all cell lines investigated show higher invading activity in vitro than monolayer-derived cells. (** p<0.01, * p<0.05).

In analogy to this observation, we asked whether CSC derived of spheroid cultures of HNSCC display a similar invasive potential. By using a Matrigel invasion chamber, we compared the invading capacity of cells either raised in spheroid or monolayer culture. SDC from all five tested cell lines showed a significantly increased invading capacity of 2.1 to 8.6 fold over the parental control (Figure 4).

### Overexpression of stemness-related genes in SDC

**Figure 5:**
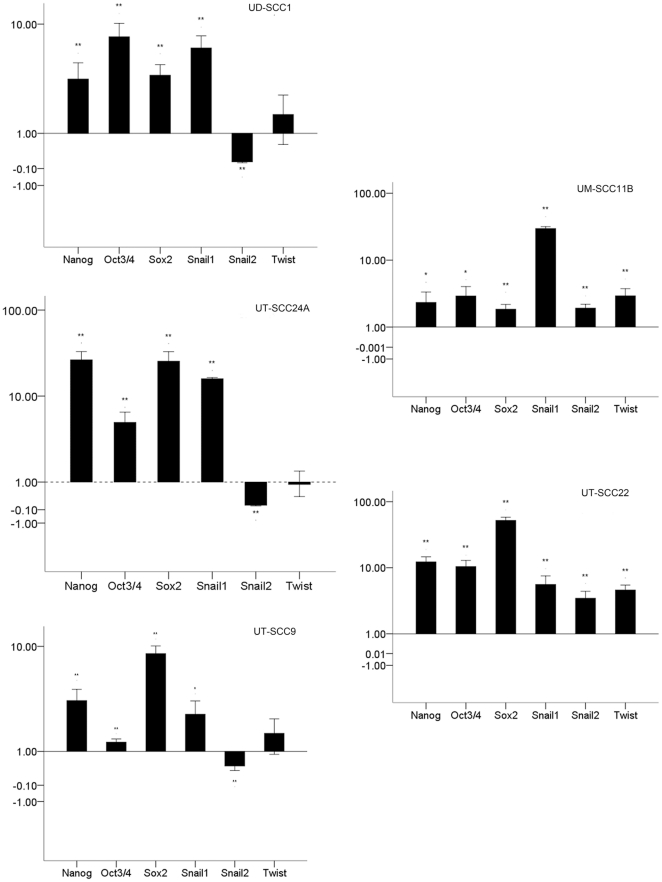
Quantitative PCR analysis of mRNA expression of stemness-related transcription factors. Messenger RNA isolated from spheroids and monolayer cultures was quantified for expression of the indicated TF. The ratio of expression in spheroid to monolayer cells is given. Significant differences were * p<0.05; ** p<0.01. The mRNA level of stemness-related TF Nanog, Oct3/4, and Sox2 were increased remarkably in spheroids. The key TF in EMT Snail1 was also increased in all spheroids but another two TF involved in EMT, Snail2 and Twist, showed an expression pattern depending on the CD44/CD24 status.

It was reported that Oct4, Sox2, and Nanog, which form a self-organized core of transcription factors (TF), maintain pluripotency and self-renewal of human embryonic stem cells [37], [38]. We wanted to know if CSC also share this feature of TF expression with embryonal stem cells (ES). For this purpose, we quantitatively compared the mRNA expression of these TF between SDC and parental monolayer-derived cells. We found that the mRNA levels of Oct3/4, Sox2, and Nanog were all significantly increased in the SDC. The highest increase was observed in UT-SCC22, where a 52-fold increase in Sox2 expression was found in spheroids. The smallest change was found in UT-SCC9, where a 1.23-fold increase in Oct3/4 expression was found in spheroids. The key TF involved in EMT, Snail1 was also significantly increased in all SDC generated from the five different HNSCC cell lines. Interestingly, two other TF involved in EMT, Snail2 and Twist, showed an expression pattern in accordance with the CD24 status. In UD-SCC1, UT-SCC9, and UT-SCC24A where most of the cells are CD44^+^/CD24^+^, the Snail2 level decreased in spheroids while Twist showed no significant change. However, in UT-SSC22 and UM-SCC11B, which were composed to over 95% of CD44^+^/CD24^−^ cells Snail2 and Twist, showed a significant increase (Figure 5). These findings implied that Snail2 and Twist expression level were correlated to the CD24 phenotype.

### SDC are more quiescent than their parental monolayer-derived counterparts

**Figure 6:**
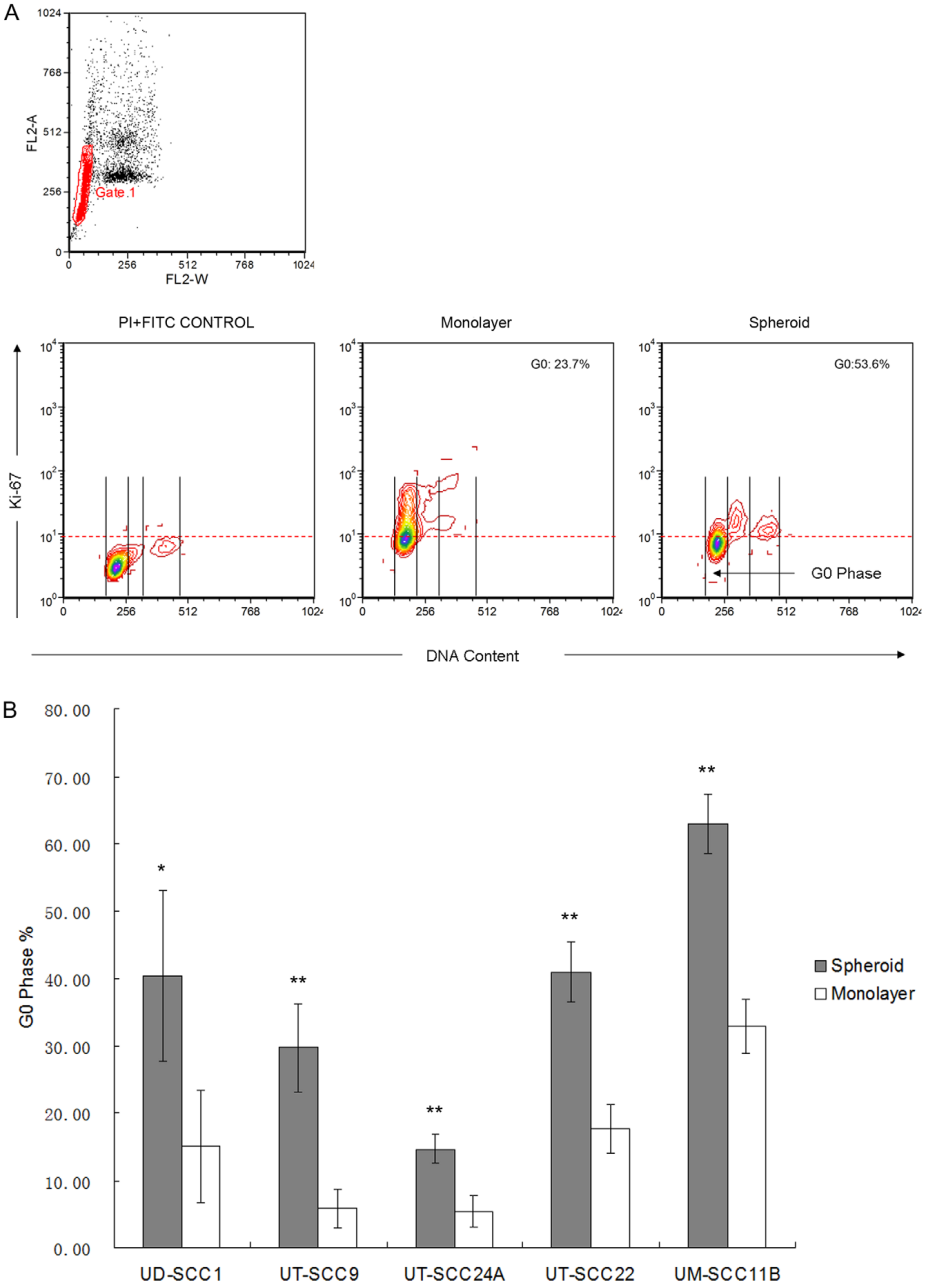
Cell cycle analysis of SDC and monolayer-derived cells by FACS analysis. (**A**) Double staining with Ki-67 and propidium iodide for the measurement of proliferation and cellular DNA content. Single cells were gated on FL2-W and FL2-A. DNA content and Ki-67 expression was evaluated by flow cytometry analysis. (**B**) Frequencies of quiescent cells in SDC as comparison to monolayer-derived cells. In all cases, the cells derived from spheroids contain a highly significant larger percentage of quiescent (G0) cells. (** p<0.01).

For cell cycle analysis by FACS, the cell lines UT-SCC24A, UT-SCC9, UD-SCC1, UT-SCC22, and UM-SCC11B grown in spheroid or monolayer culture were stained for Ki-67-FITC and DNA counterstained by propidium iodide. Cells residing in the G1/G0 peak that were negative for Ki-67 staining were regarded to be the quiescent fraction (G0 phase). In all cell lines, the proportion of cells in the G0 phase showed a highly significant increase in SDC, indicating that SDC contain a higher proportion of quiescent cells than parental monolayer-derived cells (Figure 6).

### A subpopulation of cells in spheroids have acquired features of myofibroblasts and may have undergone EMT

Myofibroblasts are components of the tumor stroma and may have a role in constructing the CSC-niche, which may help to preserve the stemness of CSC. Nevertheless, the origin of these cells remains unknown. Myofibroblasts produce several factors, which may stimulate proliferation of cancer cells and facilitate infiltration of tissue. In the literature, non-adherent spheroids generated from cancer cell lines have been shown to contain putative CSC. These spheroids could be maintained in culture for long periods, which implies that the spheroids may provide a niche suitable for the maintenance and growth of CSC. Therefore, we asked if i) myofibroblasts exist in the niche created by spheroids, ii) are these myofibroblasts derived from epithelial tumor cells through EMT, and iii) are there indications that this process is reversible?

**Figure 7:**
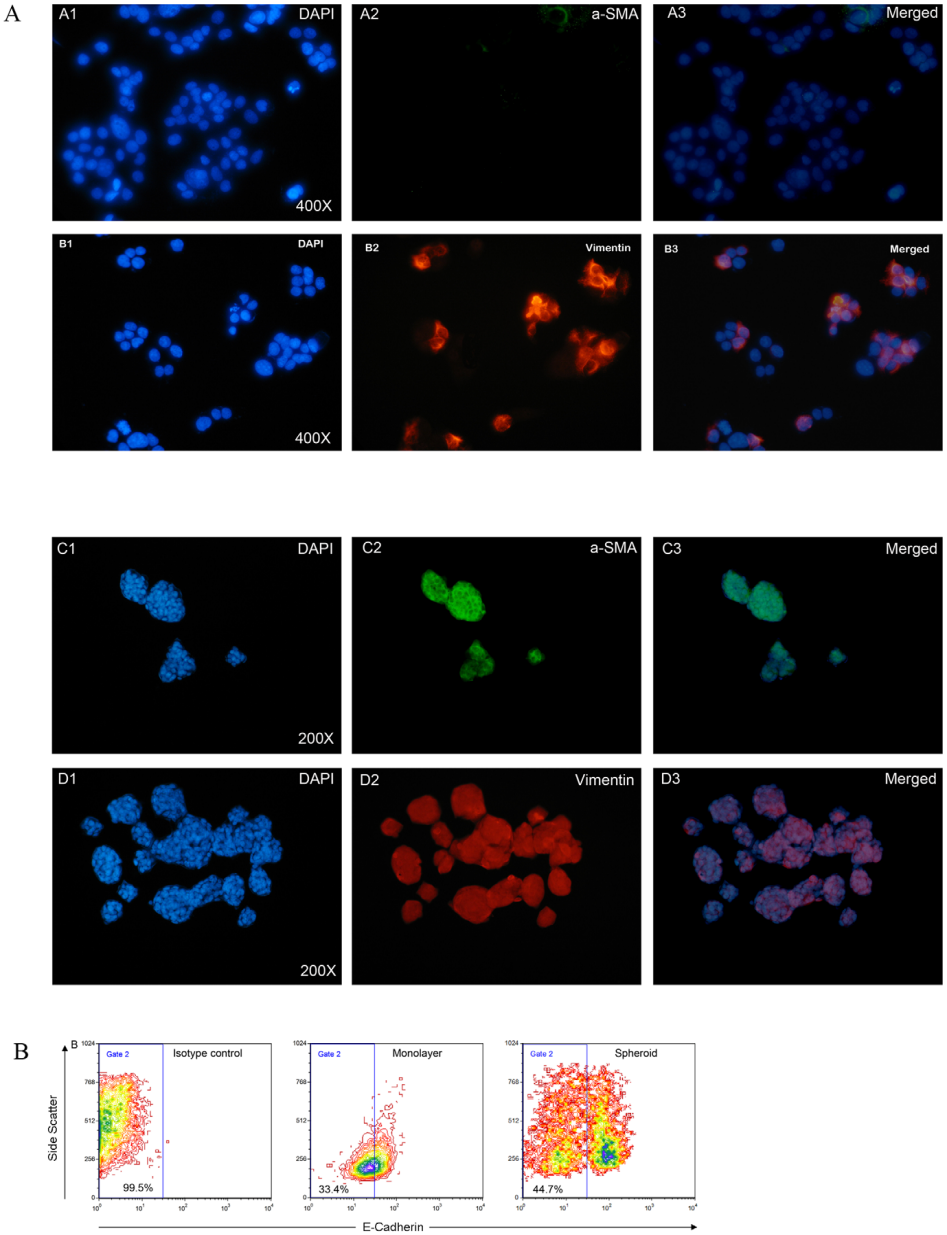
Analysis of the expression of α-SMA and Vimentin expression by immunofluorescence and E-Cadherin by FACS of monolayer versus SDC. (**A**) Immunofluorescence staining of monolayer (A1-3 and B1-3) and spheroid (C1-3 and D1-3) cultures from UT-SCC24A with antibodies directed against α-SMA (green) and Vimentin (red). All of the SDC (panel C and D) show high levels of the mesenchymal markers α-SMA and Vimentin compared to their monolayer counterparts. (**B**) E-Cadherin expression analysis by FACS. Compared to their monolayer counterparts, spheroids showed decreased E-Cadherin expression; two populations of cells derived from UT-SSC24A can be separated according to the E-Cadherin expression level, which indicates that the SDC have acquired myofibroblast characteristics by EMT.

To address this matter, we investigated the expression of myofibroblast markers Vimentin and α-SMA in SDC of 3 cell lines which have different ALDH1 expression levels in monolayer culture (low: UD-SCC1: 9.4±5.3%, medium: UT-SCC22: 16.9±4.1%, high: UT-SCC24A: 25.8±3.0%). We found that the different cell lines and SDC generated from these cell lines varied in the proportion of cells expressing Vimentin and α-SMA (Table 3). UD-SCC1 showed no α-SMA expressing cells and weak (12.5±2.5%) Vimentin expression of the cells in the monolayer culture. In contrast, UT-SCC24A (Figure 7) and UT-SCC22 monolayer-derived cells had a relatively high frequency of Vimentin and α-SMA expressing cells which indicates that these cell lines have both epithelial and mesenchymal characteristics. Interestingly, these two cell lines have a relatively high percentage of cells in the monolayer cultures that are ALDH1 positive (Table 3). Nevertheless, as spheroid cultures, all cell lines contained more α-SMA and Vimentin positive cells than the corresponding parental monolayer cell lines.

**Table 3:** Relative expression of EMT markers by monolayer and SDC-derived cells.

	α-SMA	Vimentin	E-Cadherin	ALDH1
UD-SCC1 Monolayer SDC^1^	no expression SDC: >90%	12.5±2.5% SDC: >90%	95.4±2.8% 83.5±3.1%**	9.4±5.3% 46.4±8.1%**
UT-SCC24A Monolayer SDC	6.0±1.4% SDC: >90%	36.3±5.3% SDC: >90%	62.4±4.5% 49.0±5.6%*	25.8±3.0% 42.5±8.7%*
UT-SCC22 Monolayer SDC	23.7±3.0% SDC: >90%	42.7±5.3% SDC: >90%	84.3±4.2% 63.1±5.0%**	16.9±4.1% 37.0±11.9%*

1) due to superposition cell proportions were estimated.

**P<0.01,

*P<0.05.

EMT is also accompanied by loss of cell-cell contacts to epithelial cells, characterized by a down-regulation of E-Cadherin expression. The number of E-Cadherin expressing cells in SDC and monolayers was quantified by FACS (Figure 7, Table 3). The SDC showed significantly increased proportions of E-Cadherin negative to low expressing cells compared to their monolayer-derived counterparts, indicating loss of cellular adherence in this subpopulation.

**Figure 8:**
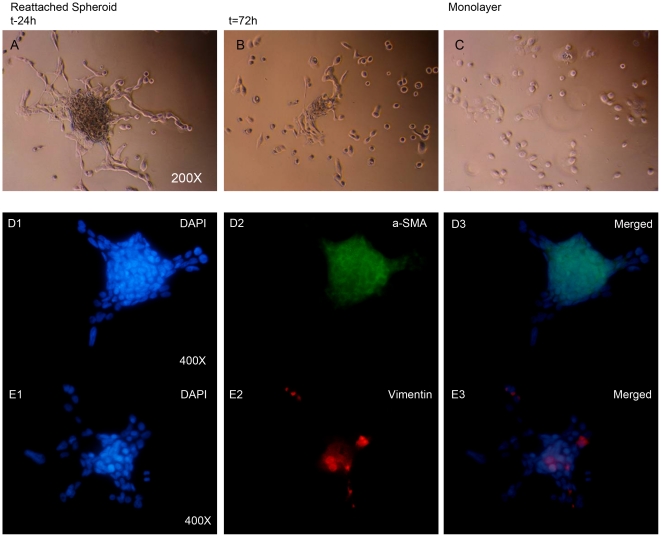
Changes in putative EMT marker expression upon reattachment of spheroids to an adhesive support. (**A–C**) Light-microscopic images with phase contrast form UD-SCC1 spheroids reattaching to cover slides during the process of re-growth to a monolayer phenotype. (A) 24 h after reattachment, some spindle-like cells grow out from adhered spheroids. (B) 72 h after reattachment, most of the attached cells' shape looks like their parental monolayer counterpart (C). (**D–E**) Indirect immunofluorescence staining of reattached spheroids after 24 h stained with DAPI (D1, E1), α-SMA (D2; green), and Vimentin (E2; red). Pictures D3 and E3 show the overlay with nuclear staining. Arrows depict outgrowing cells radiating from the spheroid. The expression of α-SMA and Vimentin decreased in outgrowing cells (arrows) after reattachment and most of the staining was confined to spheroids while most of the cells grown from spheroids were unstained.

Finally, we also tested the re-adhesion of spheroids to culture plastic. Interestingly, when the spheroids attached to the flask and began to grow out as a monolayer culture, the α-SMA and Vimentin level decreased, and most of the cells that grew out from the spheroids stained negative for these two markers (Figure 8).

## Discussion

In tumor biology many efforts have been made to explain the embryonal-like features of transformed cancer cells. The ability of CSC to rebuild the tumor from a single cell could explain many of the differences that discriminate tumor cells from differentiated somatic cells like immortality, quiescence, invasion leading to metastasis, and recurrence after treatment. Prince and colleagues [9], demonstrated that a minor population of CD44^+^ HNSCC-cells possess CSC characteristics, which could give rise to new tumors in vivo (≈5,000 cells injected). Mack and colleagues questioned the use of CD44 as a specific CSC marker in HNSCC since CD44 was abundantly expressed in most tumor cells within HNSCC (60%-100%) and therefore could not be used to distinguish normal from benign or malignant epithelia of the head and neck [10]. ALDH1 was recently shown to be a more suitable marker to identify putative CSC of HNSCC and other epithelial cancers [11], [12], [13], [14], [15]. In HNSCC, Chen et al showed that 3000 ALDH1^+^ HNSCC cells from five patients in xenotransplanted mice resulted in all cases in the generation of visible tumors 6 weeks after injection, while 10^4^ ALDH1^−^ cells failed to generate tumors. [12], [39]. We describe a method for the propagation and enrichment of ALDH1^+^ cell cultures of HNSCC cell lines. The stem cell-like characteristics of these cells were analyzed by comparing surface antigen expression and the expression of embryonal TF as well as functional characteristics of the parental monolayer cell lines. The observation that spheroid-cultures do not consist of a homogeneous cell population triggered further subanalyses that revealed a cell population with expression of EMT-markers. This may indicate that these cells have an activated EMT-program and demonstrates a potentially close relationship between CSC and cells with an activated EMT program which may have derived from the CSC.

Spheroids are three-dimensional (spherical) clusters of tumor cells grown from one or several cell clones. As compared to cell doubling times measured in monolayer culture, the rate and pattern of spheroid growth in vitro better matches that observed in tumors in vivo [40]. Anchorage independent growth has been shown to be a property shared by normal tissue cells that exhibit stem cell properties [41]. Also CSC derived from melanoma [42], breast cancer [43], and gliosarcoma [19] were able to be propagated anchorage independently and display the phenotype of nonadherent spheroids.

As exemplified in breast cancer for example, putative CSC (CD44^+^/CD24^−^) can be enriched in-vitro by isolating mammospheres from suspension cultures [43], [44]. In our study, we found the frequency of CD44^+^/CD24^−^ cells in HNSCC lines to be highly variable (from 0.5% to 97%). Moreover, they could not be enriched by spheroid culture. In contrast, ALDH1 positive cells were found to be highly enriched in spheroid cultures from all five HNSCC cell lines. If seeded at a very low densitiy, FACS sorted ALDH1^+^ cells formed significantly more colonies then the ALDH^−^ population, indicating a higher proliferative capacity of this subpopulation. Others have shown, that ALDH1^+^ or ALDH1^+^/CD44^+^/CD24^−^ cell populations isolated from HNSCC-patients have a higher tumorigenic potential then ALDH1^−^ cells even if they were CD44^+^24^−^ if xenotransplanted in mice [12].

Cancer and normal stem cells (SC) share proliferative properties of self-renewal, quiescence and expression of key transcription factors (TF). Chickarmane et al. [45] set up a computer-model to describe the key transcription factor combinations in SC and defined high levels of Oct3/4, Sox2, and Nanog. On the other hand, Ji et al. [46] compared the role of Oct3/4 and Nanog in transformed (t-hPSCs), and normal human pluripotent stem cells (hPSCs). Unlike normal SC, self-renewal and survival of t-hPSCs was found to be independent of Oct3/4 expression. In contrast, genetic knockdown of Nanog caused complete loss of self-renewal concomitantly with apoptosis in t-hPSCs. In our study, the expression of Oct3/4, Sox2, and Nanog was up-regulated in SDC in the five HNSCC derived cell lines. This implies that SDC may have stem cell properties.

Studies dealing with gene expression profiling using in vivo invasive cells or cells undergoing EMT in vitro, revealed a switch from a proliferative to an invasive phenotype. In agreement to this observation, the cell proliferation marker Ki-67 has been shown to label only a small minority of cells at the invasive front as compared to the more differentiated central part of the tumor, suggesting that non-proliferating tumor cells that have escaped the tumor mass could have metastatic potential [47]. In our study, we found that SDC had a higher proportion of Ki-67 negative cells compared to the corresponding monolayer cultures. This indicates that SDC are more quiescent than cells derived from monolayer. We also found that SDC have an increased invasion capacity, which implies that these cells may have a higher ability to metastasize.

Myofibroblasts are a kind of mesenchymal cell playing a key role in tumor invasion and metastasis and therapeutic response. They can produce several factors which may stimulate proliferation of cancer cells and facilitate their infiltration [31]. However, the origin of these myofibroblasts is not clear. EMT has been reported to play an important role in initiating CSC [29]. In this study, we demonstrated that the myofibroblast-like cells are a subpopulation of SDC. These spheroids derived exclusively from cloned tumor cells. It can therefore be concluded that the myofibroblasts could only emerge from epithelial cancer cells by altering the epithelial to a mesenchymal phenotype through EMT. More importantly, as we found, that over 90% of SDC stained positive for α-SMA and Vimentin while the percentage of ALDH1^+^ cells ranged from 37% to 46.4%. A significant decrease of E-Cadherin expression could be demonstrated in SDC, indicating a reduction of cell-cell contact in spheroid cultures. These marker expressions were reversible under culture conditions, where spheroids could adhere and cells grew radially out to form a monolayer. Taken together, these data indicate that spheroid cultures contain at least two populations that at their extremes may have either CSC- or mesenchymal characteristics, while many cells are in transition between these phenotypes. We believe that cells derived from spheroids, which had undergone a transition to a mesenchymal phenotype, maintained CSC-characteristics such as expressing a high level of the CSC marker ALDH1, an increased proportion of G0 phase cells, increased invading capacity, and elevated mRNA levels of stem cell-related transcription factors such as Nanog, Sox2, and Oct3/4.

In summary, in this study we provide an anchorage independent culturing method of HNSCC cell lines suitable for the enrichment of cells with cancer stem cell characteristics and of cells undergoing EMT. Whether this method would select, starting from non-manipulated human HNSCC samples, the tumorigenic cells remains to be demonstrated. Functionally, SDC showed higher invading capacity than monolayer cells. FACS sorted ALDH1^+^ cells showed higher colony forming ability than ALDH1^−^ cells. Our findings may prove useful to investigate the phenomenon of EMT in vitro and thus support in vivo studies investigating the role of CSC and EMT in the spread of HNSCC.

## Materials and Methods

### Cell lines

The HNSCC cell line panel was composed of UD (University of Düsseldorf, Henning Bier) -SCC 1, UM (University of Michigan, Tom Carey) -SCC -11B and UT (University of Turku, Reidar Grenman) -SCC -9, -22, -24A [48], [49]. The suffixes A, B, and C indicate cell lines derived from the primary tumor (A), metastatic (B) or recurrent (C) disease. These immortal HNSCC lines have been in culture from primary tumor for approximately 40 to 60 generations.

### Suspension culture for spheroid formation

Adherent monolayer cells were grown in normal 25 cm^2^ culture flasks (TPP, Switzerland) in DMEM (Gibco, Paisley, UK) supplemented with GlutaMAXTM-I, 1x containing 10% heat inactivated FCS and 1% Penicillin/Streptomycin, until confluency. Cells were washed with PBS twice and detached using Trypsin/EDTA solution. The reaction was stopped by adding complete culture medium. After 5 minutes, cells were resuspended in serum-free Quantum 263 medium (Biochrom AG, Berlin, Germany), supplemented with 10 ng/ml EGF and 10 ng/ml bFGF (Biochrom).

To generate spheroids, single cells were plated in Corning* Ultra-Low Attachment plates (Fisher Scientific, Loughborough, UK) at a specific density of 2×10^4^ cells/ml. Cells were kept in the incubator at 37°C in humidified atmosphere with 5% CO_2_ content. At day three, half of the medium was replaced. After 5 to 7 days, representative pictures were taken and spheroids were collected by filtration through a 40 µm mesh for the following experiments.

### Multicolor flow-cytometric (FACS) analysis and sorting

The identification of aldehyde dehydrogenase 1 (ALDH1) activity from spheroid-derived and HNSCC monolayer cells was conducted by using the ALDEFLUOR assay (StemCell Technologies, Durham, NC, USA). Spheroids were collected using a 40 µm mesh and disaggregated into single cells by Trypsin/EDTA digestion for 10 min followed by 20 times up- and down pipetting using a 1000 µm pipette tip. The single cell suspension was washed twice in PBS buffer then suspended in ALDEFLUOR assay buffer containing ALDH substrate (BAAA, 1 mol/l per 1×10^6^ cells) and incubated for 40 min at 37°C. As a negative control, for each sample of cells an aliquot was treated with 50 mmol/l diethylaminobenzaldehyde (DEAB), a specific ALDH inhibitor.

Next, for cell surface antigen phenotyping, cells were resuspended in 100 µl Aldefluor incubation buffer then stained with 20 µl anti-CD24-PE (BD bioscience, San Jose, CA, USA), 20 µl anti-CD44-APC (BD bioscience) and 5 µl 7-AAD (BD bioscience) or 5 µl anti-E-Cadherin (Biolegend, San Diego,CA, USA) per 10^6^ cells. The cells were then incubated at 4°C for 15 min in the dark. Following incubation, cells were washed once with cold FACS buffer.

For FACS sorting, cells were resuspended in PBS buffer at 1×10^7^ cells per ml and separated on an Aria cell sorter (BD Biosciences). The sorted cells could not be assessed for purity by reanalysis due to the low numbers of cells obtained. The sorting gates were established, using as negative controls the cells treated with DEAB.

### Clone formation assay

After FACS sorting, the ALDH1^+^ and ALDH1^−^ cells were inoculated to 6-well plates at a density of 1000 cells/ml in DMEM medium supplemented with 10% FBS. Cells were cultivated under standard culture conditions for 2 weeks and then the clone numbers were counted microscopically.

Spheroid culture is one of the three-dimensional culture systems that have been reported. After FACS sorting, ALDH1^+^ and ALDH1^−^ cells were inoculated into Ultra-low attachment 96-well plates (Fisher Scientific, Loughborough, UK) by using the limiting dilution method. All wells were examined carefully under microscopical vision and the wells with more than one cell or no cells were excluded from further analysis. Cells were resuspended in serum-free Quantum 263 medium (Biochrom AG, Berlin, Germany), supplemented with 10 ng/ml EGF and 10 ng/ml bFGF (Biochrom). Fresh medium was added every week. After 3 weeks, the cells contained in each well were counted and photographed.

### Quantitative real-time PCR

Total RNA was extracted by using a Qiagen RNeasy kit (Qiagen, Hilden Germany), then converted to cDNA with the Omniscript First-Strand synthesis system (Qiagen) using random primers (Qiagen). qRT-PCRs were carried out using ABI Power SYBR Green mix (ABI, Applied Biosystems Inc, Foster City, CA, USA) and run on a BioRad Chromo 4 (BioRad, München, Germany). Reactions were carried out in Triplicate with RT controls, the gene of Ribosomal protein HL32 was used as a reference gene [50], and data were analyzed using the modified delta delta Ct method. Primer sequences are listed in table 4.

**Table 4:** Primer sequences used for RT-PCR in TF expression analysis.

Transcript name	Forward primer sequence	Reverse primer sequence
SNAIL1	GGCGCACCTGCTCGGGGAGTG	GCCGATTCGCGCAGCA [51]
Nanog	AATACCTCAGCCTCCAGCAGATG	TGCGTCACACCATTGCTATTCTTC
SNAIL2	GGGGAGAAGCCTTTTTCTTG	TCCTCATGTTTGTGCAGGAG
Twist1	GGAGTCCGCAGTCTTACGAG	TCTGGAGGACCTGGTAGAGG
Oct3/4	GACAGGGGGAGGGGAGGAGCTAGG	CTTCCCTCCAACCAGTTGCCCCAAAC
Sox2	GGGAAATGGGAGGGGTGCAAAAGAGG	TTGCGTGAGTGTGGATGGGATTGGTG
hL32(reference)	AGCTCCCAAAAATAGACGCAC	TTCATAGCAGTAGGCACAAAGG [50]

### Invasion assay

Cells cultivated in a monolayer or in a spheroid-culture were separated into single cells by careful trypsin digestion and resuspended in 1% BSA-DMEM culture medium. Cells (5×10^4^) were seeded into the upper compartments of BD BioCoat™ Matrigel Invasion Chambers (BD Bioscience), and DMEM supplemented with 10% FBS was added to the lower compartment according to manufacturer's instructions. The invasion chamber was kept for 24 h under standard culture conditions. After incubation, the non-invading cells were removed from the upper surface of the membrane by gentle scrubbing, and the cells on the lower surface of the membrane were stained with crystal violet. Cell counting was facilitated by photographing the membrane through the microscope and 3 fields per membrane of triplicate membranes were counted under 200× magnification (Axiovert, Axiovision, Zeiss, Germany).

### Cell cycle analysis and Ki-67 staining

The parental cells and spheroids were harvested, dissociated into single cells and resuspended in 70% ethanol by adding ethanol at −20°C dropwise while vortexing. The cells were stored at −20°C overnight then washed with PBS, centrifuged and 1 µg FITC-conjugated mouse anti-human Ki-67 (DAKO, Glostrup, Denmark) was added to the cell pellet and incubated for 20 min at room temperature. Finally, cells were resuspended in 1 ml of a cocktail containing propidium iodide (2 µg/ml) and RNAse (100 µg/ml) (Fermentas Life Science, St. Leon-Rot, Germany) and stained for 30 min followed by FACS analysis.

### Immunofluorescence

Indirect immunofluorescence staining was performed on spheroids and on parental adherent cultures of cancer cells for α-SMA and Vimentin. Spheroids were collected by filtering the suspension culture medium through a 40 µm mesh (BD bioscience), and then whole spheroids were cytospined (1500 rpm, 5 min) onto slides. Cells were fixed in 4% paraformaldehyde then permeabilized with 0.1% Triton in PBS solution for 10 min. First, mouse anti-human Vimentin (1∶50 from stock; Santa Cruz Biotechnology, Heidelberg, Germany) and mouse anti-human α-SMA diluted (1∶50 from stock; Dako) antibodies were added and incubated overnight at 4°C. Then the secondary antibody, Alexa488-conjugated goat anti-mouse IgG (Invitrogen, Karlsruhe, Germany) or Alexa568-conjugated donkey anti-mouse IgG (1∶200 from stock) (Invitrogen), was added and incubated for 60 min at room temperature. Counterstaining was performed by using DAPI (1 µg/ml Roche, Mannheim, Germany). For quantification, the average percentages of positive cells from three experiments were estimated. Due to superposition in the case of SDC, cell proportions are less reliable. Therefore, no significance was calculated for Vimentin and α-SMA.

## Supporting Information

Figure S1 **Single ALDH1^+^ cells proliferated and formed a spheroid significantly more often then ALDH1^−^ cells.** (**A**) FACS-sorting strategy for SDC of UD-SCC1. Representative pictures of the cell line UD-SCC 1 are shown. (**B**) ALDH1^−^ or ALDH1^+^ single cells were cultured in 96-well ultra-low attachment plates. Three weeks after inoculation, 19.3% of ALDH1^+^ SDCs formed spheroids while ALDH1^−^ cells failed to form spheroids except for one case (p<0.01).
(TIF)
