# Supplementary figures and images for: Evidence for Epithelial-Mesenchymal Transition in Cancer Stem Cells of Head and Neck Squamous Cell Carcinoma
Source: PLoS One. 2011 Jan 27;6(1):e16466. doi: 10.1371/journal.pone.0016466 (PMC3029362; doi:10.1371/journal.pone.0016466)

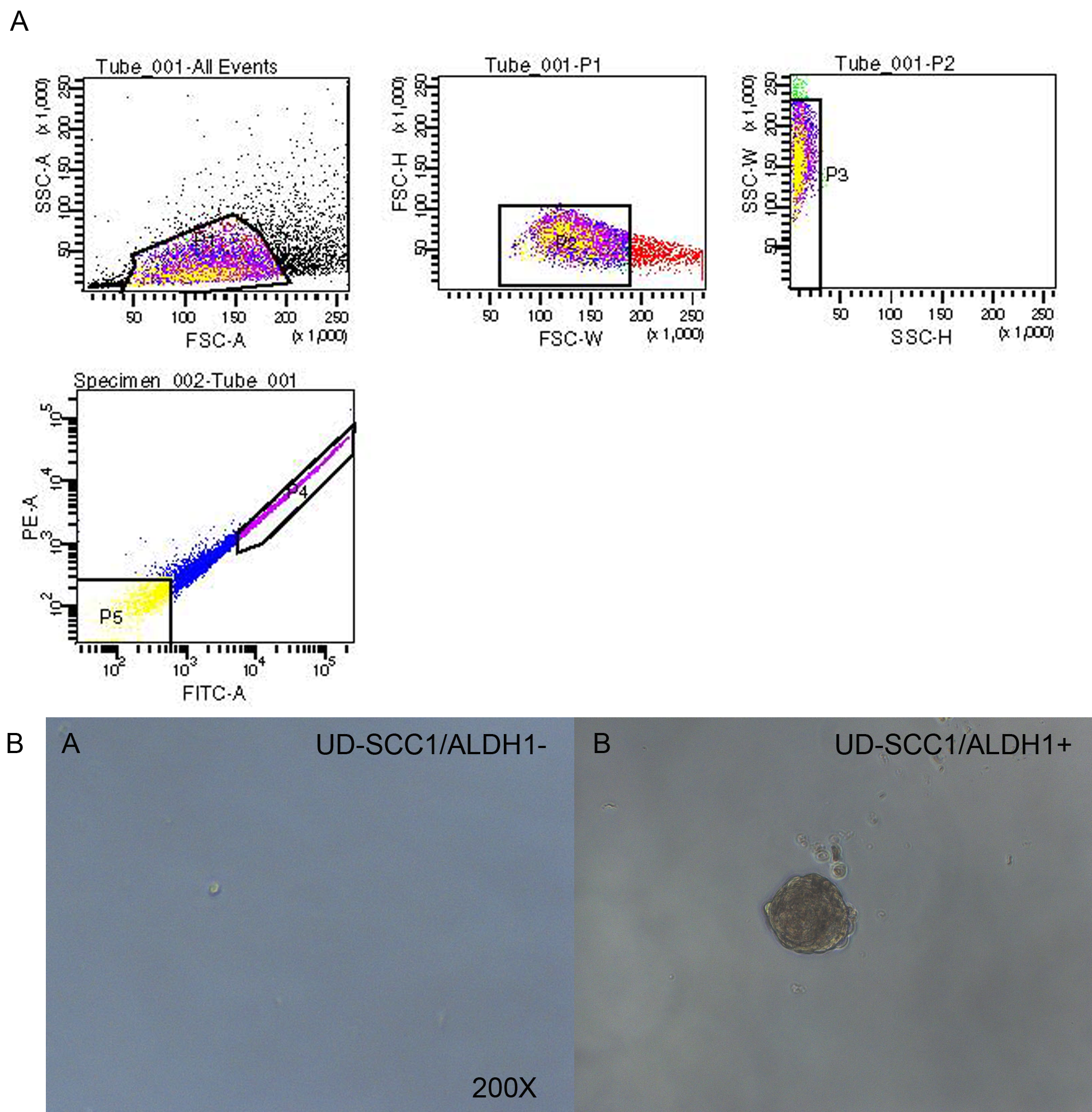

Supplement: Figure S1 — Single ALDH1+ cells proliferated and formed a spheroid significantly more often then ALDH1− cells. (A) FACS-sorting strategy for SDC of UD-SCC1. Representative pictures of the cell line UD-SCC 1 are shown. (B) ALDH1− or ALDH1+ single cells were cultured in 96-well ultra-low attachment plates. Three weeks after inoculation, 19.3% of ALDH1+ SDCs formed spheroids while ALDH1− cells failed to form spheroids except for one case (p<0.01). (TIF) [file pone.0016466.s001.tif]
